# Six-Year Remission With No Relapse After Four-Time Weekly Rituximab Only for Bilateral Ocular Adnexal Follicular Lymphoma

**DOI:** 10.7759/cureus.88945

**Published:** 2025-07-28

**Authors:** Toshihiko Matsuo, Takehiro Tanaka, Nobuharu Fujii

**Affiliations:** 1 Ophthalmology, Graduate School of Interdisciplinary Science and Engineering in Health Systems, Okayama University, and Okayama University Hospital, Okayama City, JPN; 2 Department of Pathology, Graduate School of Medicine, Dentistry, and Pharmaceutical Sciences, Okayama University, Okayama, JPN; 3 Division of Transfusion and Cell Therapy, Department of Hematology and Oncology, Okayama University Hospital, Okayama City, JPN

**Keywords:** claustrophobia, extranodal marginal zone b-cell lymphoma mucosa-associated lymphoid tissue (malt) type, fluorodeoxyglucose positron emission tomography, follicular lymphoma, magnetic resonance imaging, mucosa-associated lymphoid tissue (malt) lymphoma, ocular adnexa, orbital mass, radiotherapy, rituximab

## Abstract

Follicular lymphoma mostly takes an indolent course, and thus, observation with watchful waiting is a main therapeutic strategy. Recent long-term studies suggest earlier treatment with rituximab monotherapy may benefit patients by delaying the need for treatment in the later phase of exacerbation. In this study, we reported a patient with bilateral orbital follicular lymphoma who received four-time weekly rituximab monotherapy as an induction therapy only and maintained the remission for 5 years with no treatment. The patient was a 51-year-old woman who developed a right upper orbital mass and was diagnosed with follicular lymphoma grade 1 by the excisional biopsy. Two years later, at the age of 53 years, she developed a left lacrimal gland mass and underwent excision. The pathological diagnosis was follicular lymphoma grade 1. She did not have any other systemic lesions by fluorodeoxyglucose positron emission tomography. At the age of 54 years, she developed a new mass on the nasal side of the right orbit and underwent weekly rituximab monotherapy (375 mg/m^2^) four times a month, leading to the reduction of the mass in 3 months. Two high uptake sites on the temporal and nasal side of the right superior orbit by fluorodeoxyglucose positron emission tomography disappeared one year later at the age of 55 years. She was followed with no treatment for 6 years until the age of 60 years at the latest visit. In case of a local orbital relapse, local radiotherapy would be the standard, but rituximab monotherapy as an induction therapy only was chosen in the present patient. Rituximab monotherapy in place of local radiotherapy would be a treatment option for orbital follicular lymphoma.

## Introduction

Lymphoma and other related inflammatory diseases often develop bilaterally in the orbit. The orbital content, called ocular adnexa, serves as a supporting tissue for the eyeball and is specified as the conjunctiva and eyelid, lacrimal gland and sac, and extraocular muscles. A predominant histopathological type of lymphoma in the ocular adnexa is extranodal marginal zone B-cell lymphoma, mucosa-associated lymphoid tissue (MALT) type, in brief, MALT lymphoma [1-4], but there is rare involvement with other types of lymphoma such as follicular lymphoma, mantle cell lymphoma, and diffuse large B-cell lymphoma [5-7]. The ocular adnexa, especially the lacrimal glands, are also involved with inflammatory diseases such as sarcoidosis, immunoglobulin G4 (IgG4)-related disease, and other rare diseases like Kimura disease [8-12]. It should be noted that these inflammatory diseases may serve as a predisposition or a background for developing lymphoma later. Under the circumstances, pathological examinations of orbital and ocular adnexal lesions are mandatory to establish a correct diagnosis and hence, to choose an appropriate treatment plan.

Follicular lymphoma is a rare histopathological type of lymphoma that involves the orbit and ocular adnexa, as stated above [13,14]. Follicular lymphoma in the ocular adnexa has a greater tendency to have infiltration in other areas of the body, compared with MALT lymphoma, and thus, a treatment plan should be considered on the basis of probable systemic infiltration. In general, a treatment strategy for follicular lymphoma has been watchful waiting, but recently, early intervention with rituximab monotherapy may be better in delaying the progression of the disease in the later phase [15]. In this study, we present a patient who developed orbital follicular lymphoma first on the right side and 2 years later on the left side. Weekly rituximab monotherapy, four times only for an active residual and recurrent lesion on the right side, led to the subsidence and no disease in the further 5 years.

## Case presentation

A 51-year-old woman noticed right upper eyelid swelling for several weeks. She had been taking valsartan 40 mg daily for hypertension and atorvastatin 10 mg for dyslipidemia for 10 years. In her past history, she had experienced hyperthyroidism after the delivery at the age of 30 years and had taken an antithyroid drug for 10 years. At the initial visit, the best-corrected visual acuity in decimals was 1.5 in both eyes, and the intraocular pressure was 16 mmHg in both eyes. She had no diplopia and showed normal eye movements. The ocular media and fundus in both eyes were normal. She showed mild proptosis on the right side, and a mass was palpable along the superior orbital bone rim. The other physical examinations were normal. She did not have lymphadenopathy, fever, general fatigue, or weight loss. The complete blood cell counts and blood chemistry, including thyroid hormones (free T3, free T4, and thyroid-stimulating hormone), as well as urinalysis, were also normal (Table 1). Serum thyroid-stimulating autoantibody was negative. Serum soluble interleukin-2 receptor (sIL-2R) was normal at 264 U/mL (normal range: 156.6-474.5 U/mL), and immunoglobulin G4 (IgG4) was normal at 48.3 mg/dL (normal range: 4.8-105.0 mg/dL).

**Table 1:** Blood examinations at age 51 with right orbital mass, at age 53 with left orbital mass, and at the latest visit at age 60 n.d.: not determined Normal ranges at the in-house laboratory were for the time at age 60 years.

	Normal Range	At 51 Years	At 53 Years	At 60 Years
Red blood cells (× 10^6^/µL)	3.86-4.92	4.28	4.58	4.48
Platelets (× 10^3^/µL)	158-348	294	330	265
White blood cells (× 10^3^/µL)	3.30-8.60	5.60	5.74	6.44
Neutrophils (%)	40.0-70.0	60.7	49.2	51.2
Lymphocytes (%)	16.5-49.5	33.3	45.0	43.2
Monocytes (%)	2.0-10.0	3.7	3.6	3.5
Eosinophils (%)	0.0-8.5	1.8	1.7	1.8
Basophils (%)	0.0-2.5	0.5	0.5	0.4
Hemoglobin (g/dL)	11.6-14.8	13.8	14.4	14.1
Hematocrit (%)	35.1-44.4	40.9	44.9	44.5
Total protein (g/dL)	6.6-8.1	6.7	7.0	6.3
Albumin (g/dL)	4.1-5.1	3.9	4.1	3.6
Lactate dehydrogenase (LD) (U/L)	124-222	179	183	201
Aspartate aminotransferase (AST) (U/L)	13-30	14	12	14
Alanine aminotransferase (ALT) (U/L)	7-23	14	12	12
γ-glutamyl transferase (γ-GT) (U/L)	9-32	14	14	15
Total bilirubin (mg/dL)	0.40-1.50	0.74	0.67	0.55
Urea nitrogen (mg/dL)	8.0-20.0	12.0	15.9	18.2
Creatinine (mg/dL)	0.46-0.79	0.76	0.75	0.81
Estimated glomerular filtration rate (eGFR) (mL/min/1.73 m^2^)	60 or greater	62.6	63.2	55.7
Uric acid (mg/dL)	2.6-5.5	4.7	4.6	5.7
Total cholesterol (mg/dL)	142-248	219	326	270
Postprandial blood glucose (mg/dL)	<140	117	82	n.d.
C-reactive protein (CRP) (mg/dL)	<0.15	n.d.	n.d.	0.04
Soluble interleukin-2 receptor (sIL-2R) (U/mL)	156.6-474.5	264	206	266.3
Immunoglobulin G4 (IgG4) (mg/dL)	4.8-105.0	48.3	73.1	n.d.

**Figure 1:**
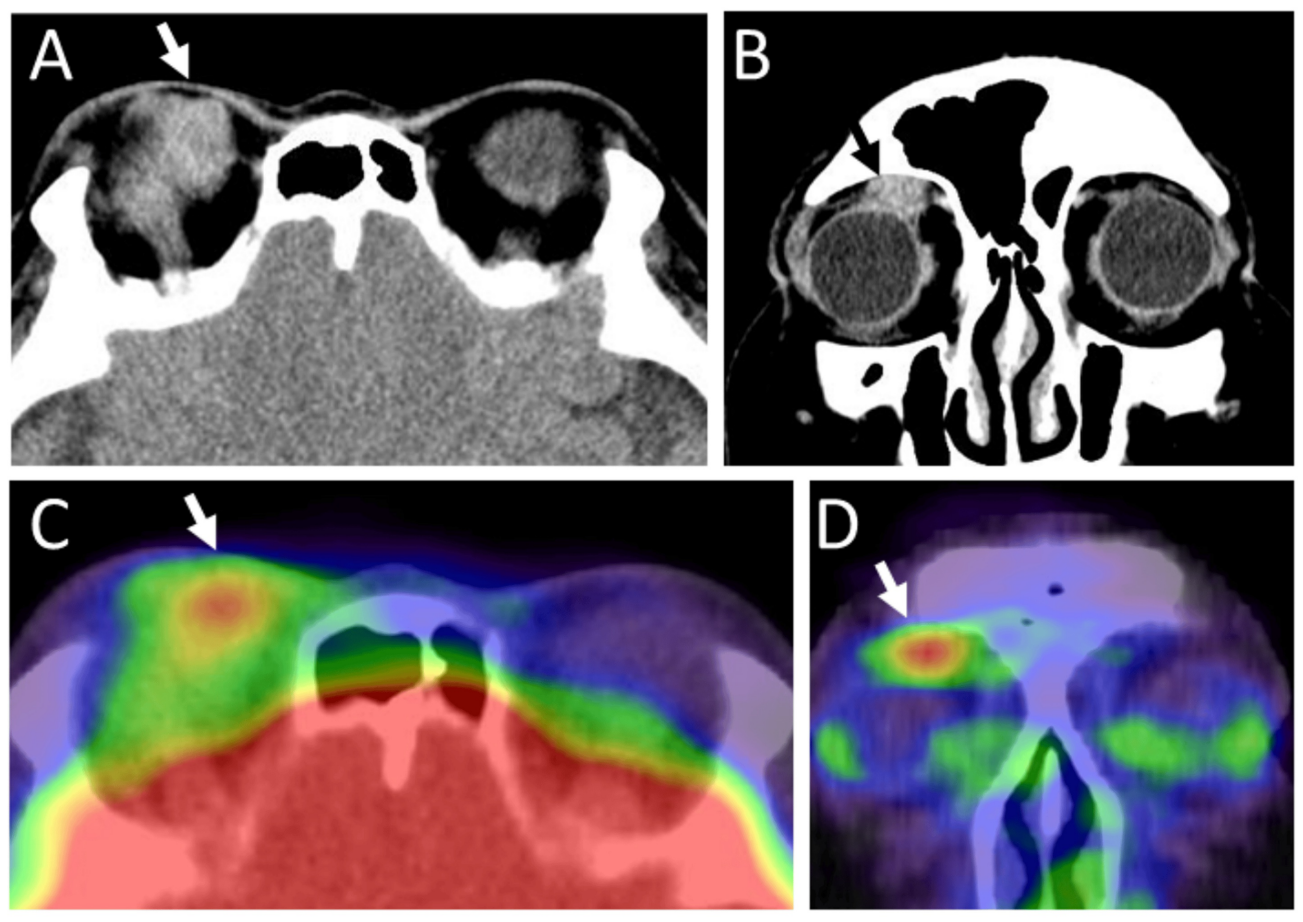
Computed tomography scans and positron emission tomography at age 51 Computed tomography scans in axial image (A) and coronal image (B) at age 51, showing an irregular-shaped mass (arrows) in the superior nasal orbit above the eyeball. Fluorodeoxyglucose positron emission tomography in axial image (C) and coronal image (D), showing only an abnormal uptake site (arrows) in the superior nasal orbit.

**Figure 2:**
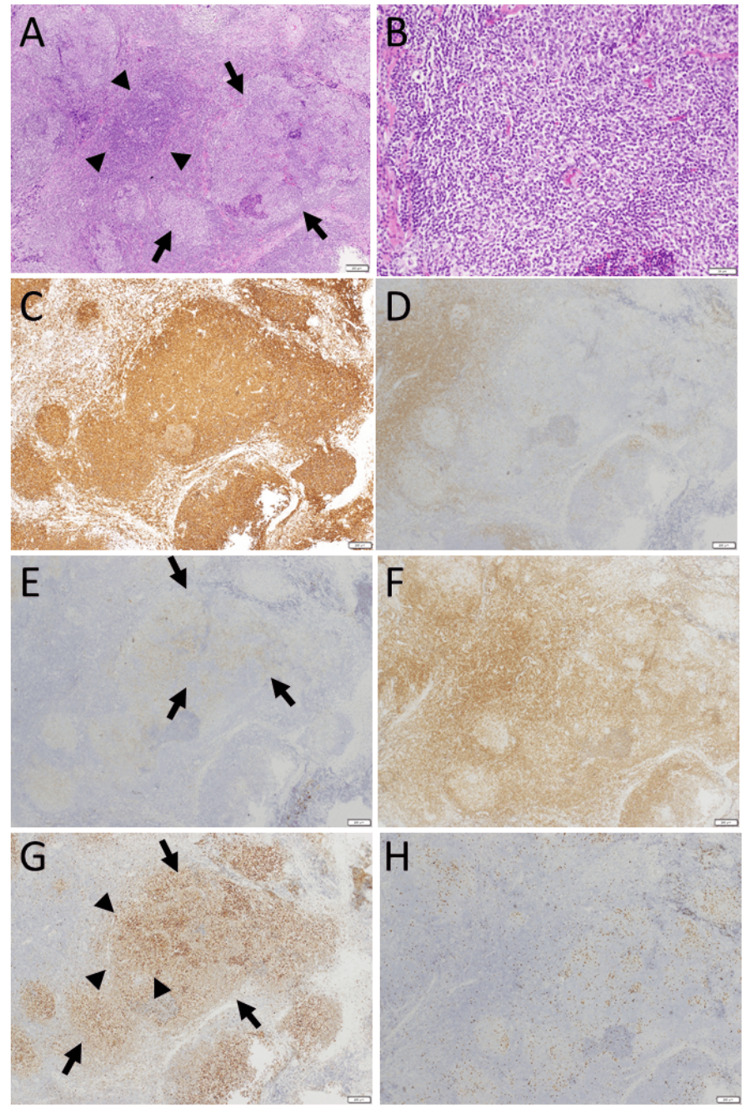
Pathology images of the right orbital mass at age 51 Excisional biopsy of the right orbital mass at age 51, showing medium-sized lymphoid cells forming nodular structures like follicular centers (arrows, A) in hematoxylin-eosin stain (A: low magnification, B: high magnification). Note also the area with marginal zone-like appearance (arrowheads, A). Lymphoid cells are mostly CD20-positive B-cells (C), admixed with a small number of CD3-positive T-cells (D). Lymphoid cells are weakly positive for CD10 (E), dominantly positive for BCL2 (F), and BCL6 (G). Ki-67 labeling index is low (H). Note that lymphoid cells in follicle-like structures (arrows, A) are weakly positive for CD10 (arrows, E) and highly positive for BCL6 (arrows, G), and that lymphoid cells with a marginal zone-like appearance are also positive for BCL6 (arrowheads, G), in support of the diagnosis of follicular lymphoma. White scale bar = 200 µm in A, C-H, except for bar =50 µm in B.

Computed tomography scans showed an orbital mass, above the eyeball on the right side (Figures 1A-1B), and fluorodeoxyglucose positron emission tomography disclosed no abnormal uptake site, except for a high uptake in the right upper orbit (Figures 1C-1D). She had claustrophobia and liked to avoid magnetic resonance imaging as far as possible. Excisional biopsy of the right orbital mass showed follicular lymphoma grade 1: centrocyte or centroblast-like lymphoid cells in ill-defined geminal center-like structures (Figure 2A, 2B) were positive for CD20 (Figure 2C), negative for CD3 (Figure 2D), weakly positive for CD10 (Figure 2E), predominantly positive for BCL2 (Figure 2F) and BCL6 (Figure 2G). Ki-67 labeling index was low (Figure 2H). Bone marrow biopsy and bone marrow cell counts were normal.

**Figure 3:**
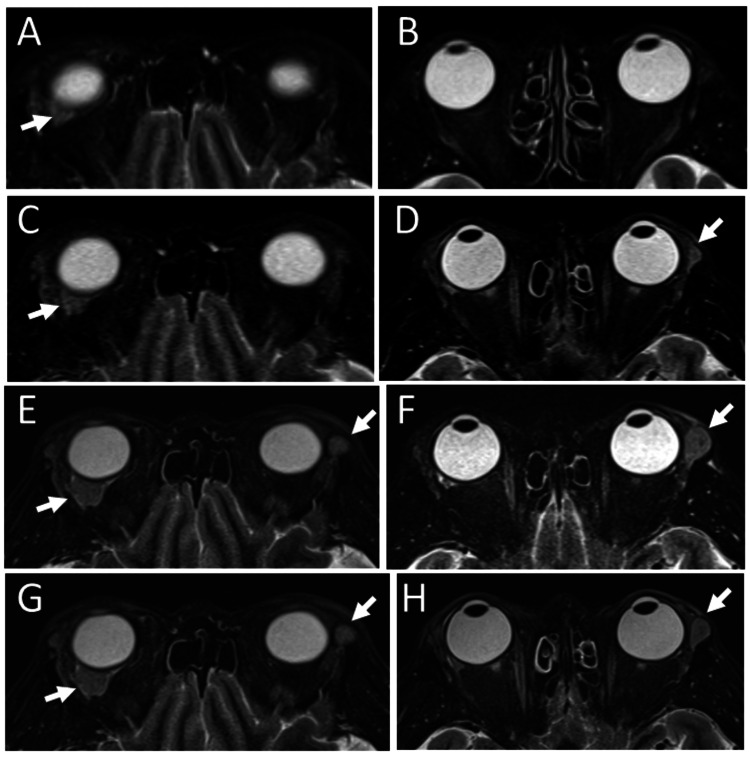
Magnetic resonance imaging at age 51 to 53 T2-weighted STIR (short tau inversion recovery) magnetic resonance imaging (A, B) 2 months later from excisional biopsy of right orbital mass at age 51, showing only a small residual mass (arrow, A) in the right upper orbit. Stable residual lesion on the right upper orbit (arrow, C) and a new mass lesion in the left superior lateral orbit (arrow, D), at age 52, 8 months later from the right excisional biopsy. The enlargement of the right superior orbital mass (arrow, E) and the left lateral orbital mass (arrow, F) 14 months from the right excisional biopsy. At age 53, just before left orbital mass excision, 20 months from the right orbital excisional biopsy, the same mass lesions are detected in bilateral orbits (arrows, G, H).

**Figure 4:**
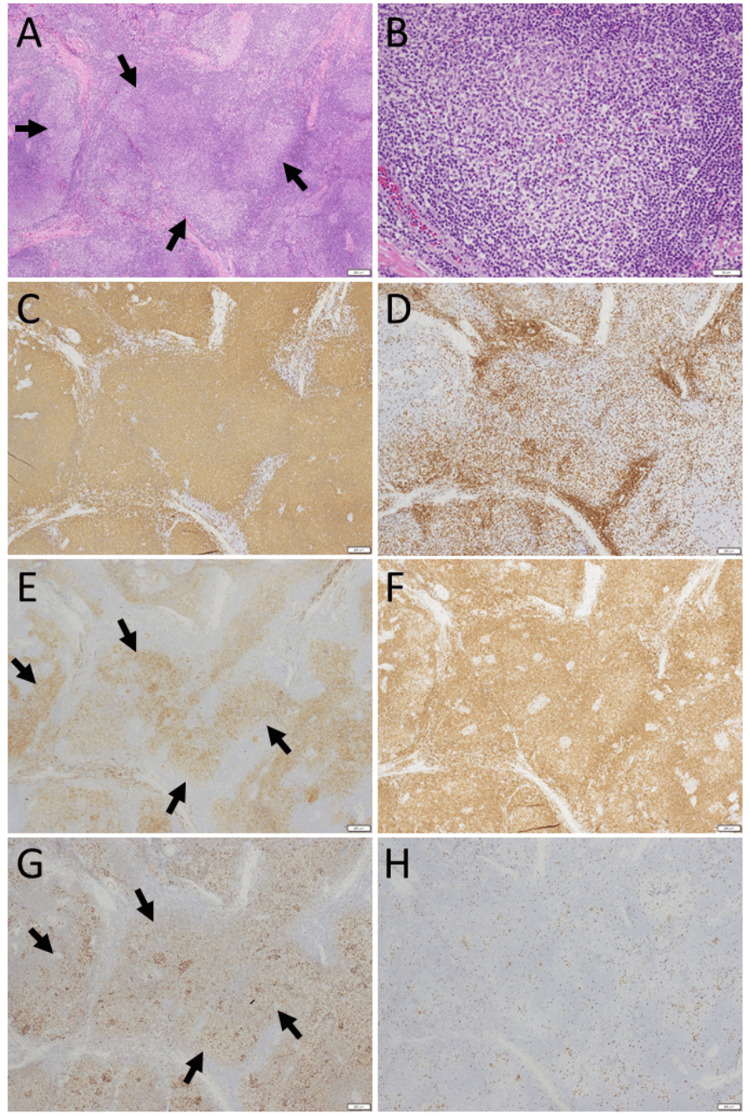
Pathology images of left orbital mass at age 53 Excision of the left orbital mass of the lacrimal gland at age 53, showing medium-sized lymphoid cells in neoplastic follicle-like structures (arrows, A) in hematoxylin-eosin stain (A: low magnification, B: high magnification). Lymphoid cells are mostly CD20-positive B-cells (C) with a small number of CD3-positive T-cells (D). Lymphoid cells are positive for CD10 (E), BCL2 (F), and BCL6 (G). Ki-67 labeling index is low (H). Note that lymphoid cells in follicle-like structures (arrows, A) are positive for CD10 (arrows, E) and BCL6 (arrows, G), in support of the diagnosis of follicular lymphoma. White scale bar = 200 µm in A, C-H, except for bar =50 µm in B.

She was stable with no treatment (Figures 3A-3D) until 14 months later, when magnetic resonance imaging, which she decided to undergo, showed a new orbital mass, temporal to the eyeball on the left side, in addition to the remaining mass on the right side (Figures 3E-3F). Repeat magnetic resonance imaging, 6 months later, still showed the left orbital mass (Figures 3G-3H) and thus, she underwent complete excision of the mass on the left side at the age of 53 years. The pathology was follicular lymphoma grade 1: centrocyte or centroblast-like lymphoid cells in ill-defined geminal center-like structures (Figures 4A-4B) were positive for CD20 (Figure 4C), negative for CD3 (Figure 4D), positive for CD10 (Figure 4E), BCL2 (Figure 4F), and BCL6 (Figure 4G). Ki-67 labeling index was low (Figure 4H).

**Figure 5:**
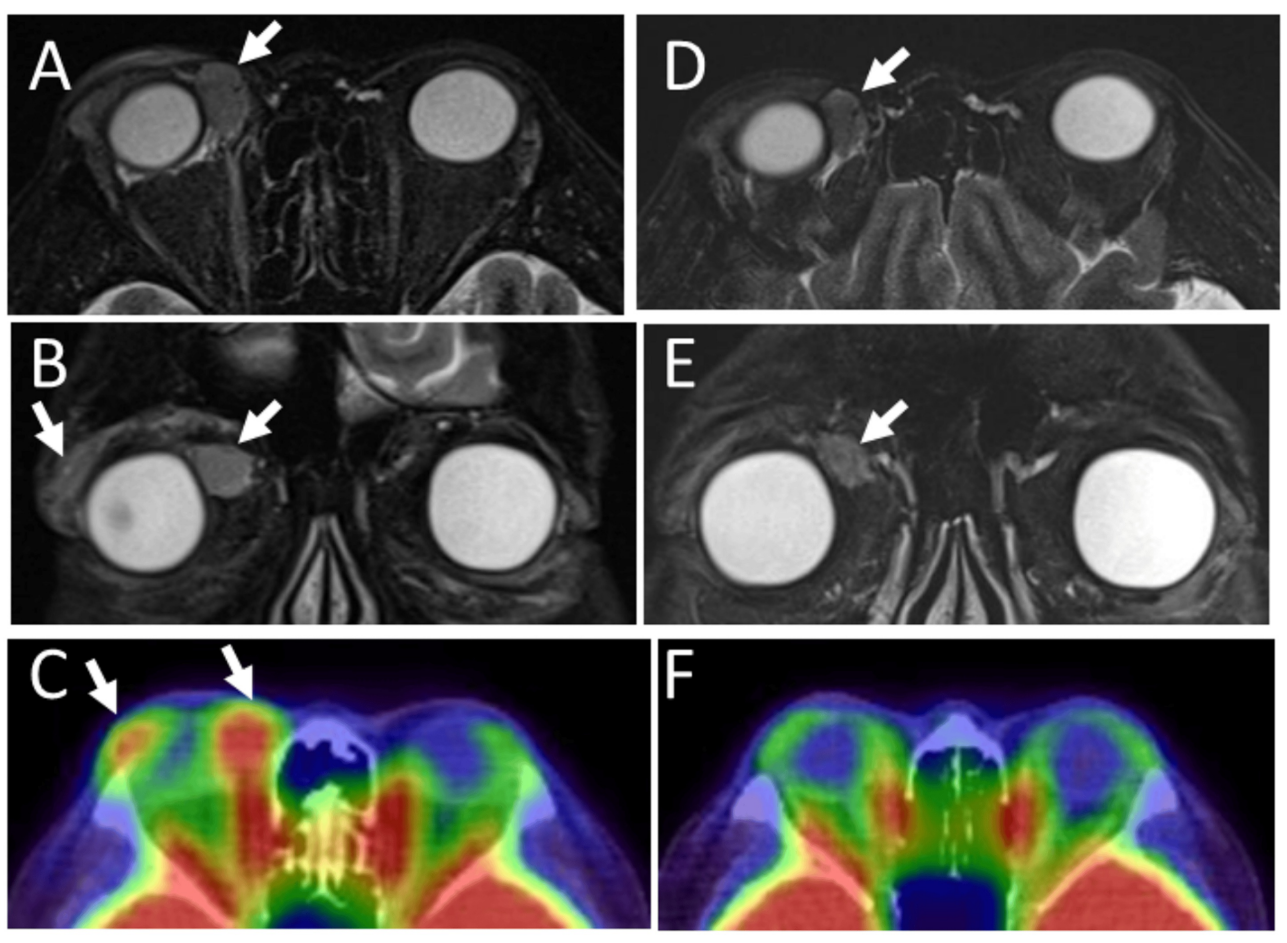
Magnetic resonance imaging and positron emission tomography at ages 54 and 55 T2-weighted STIR (short tau inversion recovery) magnetic resonance imaging at age 54, 10 months after left orbital mass excision, showing an enlarged lacrimal gland mass and a new nasal-superior mass (arrows, A, B) in the right orbit and no lesion in the left orbit (A: axial image, B: coronal image). Fluorodeoxyglucose positron emission tomography (C), showing high uptake sites (arrows) in the lacrimal gland and the nasal mass in the right orbit. T2-weighted STIR magnetic resonance imaging after four injections of weekly rituximab monotherapy in a month (D: axial image, E: coronal image). Note the diminished mass in the right nasal orbit (arrows, D, E) and no lesions in the bilateral lacrimal glands. Positron emission tomography (F) 7 months after rituximab monotherapy, showing no abnormal uptake in bilateral orbits.

One year later, at the age of 54 years, a mass lesion was palpable on the nasal-superior edge of the right orbit, and magnetic resonance imaging showed a new mass on the nasal side of the right orbit (Figures 5A-5B). She thus underwent weekly rituximab monotherapy (375 mg/m^2^) four times in a month, leading to the reduction of the mass in 3 months, as shown by magnetic resonance imaging (Figure 5D-5E). Two high uptake sites on the temporal and nasal side of the right superior orbit by fluorodeoxyglucose positron emission tomography (Figure 5C), which was noted before the rituximab monotherapy disappeared 10 months later at the age of 55 years (Figure 5F). She was followed with no treatment for 6 years until the age of 60 years at the latest visit. Magnetic resonance imaging was not done because of her claustrophobia. Serum soluble interleukin-2 receptor remained in the normal range and did not show a tendency to rise throughout the course (Table 1). She showed no signs or symptoms, including lymphadenopathy.

## Discussion

According to the Follicular Lymphoma International Prognostic Index (FLIPI), she did not fit with any factors: age (61 years or older), stage (III or IV), hemoglobin (<12 g/dL), the number of lymphadenopathy (>5), and serum lactate dehydrogenase (over the upper limit). She also had no bone marrow involvement, as a factor which is designated in a modified version 2 of FLIPI. The isolated mass lesion in the orbit with the diagnosis of follicular lymphoma is usually treated with local radiotherapy [13,14,16]. She chose observation even though she had a residual lesion in the right orbit. Two years later, she developed a mass lesion which was probably derived from the lacrimal gland in the left orbit, on the opposite side to the initial right orbital mass. One year after the almost complete excision of the left orbital mass, the right orbital mass gained activity again. This time, she chose four-time weekly rituximab monotherapy, which led to remission in one year, as evidenced by fluorodeoxyglucose positron emission tomography. She maintained the clinical remission for 5 years until the latest visit without any treatment.

The pathological diagnoses of bilateral orbital masses, which developed in the interval of 2 years, were follicular lymphoma grade 1. Watchful waiting, which means active surveillance, is a treatment strategy for grade 1 follicular lymphoma, which takes an indolent course in terms of years. Rituximab monotherapy has been evaluated as an induction therapy and as a maintenance therapy in follicular lymphoma with low tumor burden [15]. In the present patient, four-time weekly intravenous rituximab monotherapy was done as an induction therapy, and the maintenance therapy was not done, based on the clinical remission, which was evidenced by no abnormal uptake in bilateral orbits 7 months later on fluorodeoxyglucose positron emission tomography. Afterwards, in 5 years, she remained free of symptoms and signs with no treatment. A major limitation in this patient was the fact that magnetic resonance imaging was not done as a follow-up examination in the recent 5 years, since we respected the patient’s claustrophobia.

In the series of 24 patients with ocular adnexal follicular lymphoma in the Danish national registry, only one patient was reported to receive rituximab monotherapy, while most patients underwent local radiotherapy and combination chemotherapy [14]. The reason for the choice of rituximab monotherapy and the outcome in that patient have not been described in detail. In the present patient, it is unique to apply rituximab monotherapy in the early phase of ocular adnexal follicular lymphoma, according to the recent long-term results in systemic follicular lymphoma with low tumor burden [15]. Rituximab monotherapy has been evaluated and recommended as an option for better quality of vision in patients with ocular adnexal MALT lymphoma, in contrast with local radiotherapy [17-19]. As far as the present patient is concerned, ocular adnexal follicular lymphoma may follow a similar path as ocular adnexal MALT lymphoma.

As regards the pathological diagnosis, the diagnosis of follicular lymphoma on the left side had no argument. In contrast, the histopathological diagnosis on the right side, which was made 2 years earlier, might be interpreted as an intermediate between follicular lymphoma and MALT lymphoma. In the previous studies, marginal zone B-cell lymphoma with follicular colonization has been reported [20-22]. On the other side of the coin, the lesion on the right side might be otherwise called follicular lymphoma with marginal zone differentiation, as reported previously [23-26]. In the sequence of events of lymphoma from the right side to the left side, follicular lymphoma would be an overall appropriate diagnosis for the right orbital mass lesion. Table 2 summarizes practical immunohistochemistry markers for follicular lymphoma, marginal zone B-cell lymphoma, and mantle cell lymphoma, which have been described in the literature [27]. In the orbital lesions on both sides of the present patient, lymphoma cells were positive for BCL6, which would be used to be differentiated from marginal zone B-cell lymphoma (Table 2). BCL2 and immunoglobulin heavy chain gene translocation t (14;18) (q23;q21) was not tested in this patient. Duodenal follicular lymphoma may have a similar feature to the present patient as an intermediate of follicular lymphoma and marginal zone B-cell lymphoma [28]. Indeed, a case report has described a patient with duodenal follicular lymphoma who had a favorable prognosis for 15 years only with rituximab monotherapy [29].

**Table 2:** Practical immunohistochemistry markers for follicular lymphoma, marginal zone B-cell lymphoma, and mantle cell lymphoma Modified from the literature [27]. Ki-67 is variably positive. +: positive in most cases; -: negative in most cases

Markers	Follicular Lymphoma	Marginal Zone B-cell Lymphoma	Mantle Cell Lymphoma
CD3	-	-	-
CD5	-	-	+
CD10	+	-	-
CD20	+	+	+
Cyclin D1	-	-	+
BCL2	+	+	+
BCL6	+	-	-
Ki-67	+	+	+

## Conclusions

The present patient in her fifties developed bilateral orbital mass lesions sequentially on the right side and then on the left side two years apart. The pathological diagnosis for bilateral orbital lesions was follicular lymphoma grade 1 in common. One year later, she developed a new orbital lesion on the right side and thus, underwent four-time weekly rituximab monotherapy sessions as an induction therapy only, and the lesion subsided in half a year. She maintained the remission for 5 years. Local radiotherapy would be the standard in case of an orbital relapse of follicular lymphoma, but rituximab monotherapy could be listed as a treatment option in orbital follicular lymphoma.
